# Supplementary material for: Farm production diversity, household dietary diversity, and nutrition: Evidence from Uganda’s national panel survey
Source: PLoS One. 2022 Dec 16;17(12):e0279358. doi: 10.1371/journal.pone.0279358 (PMC9757588; doi:10.1371/journal.pone.0279358)
Supplement: S1 Table — (DOCX) [file pone.0279358.s001.docx]

# **S 1 Table. Crops and animal species used to calculate FPD index**

**Crops for the Year 2019:**

**Top 9 grown crops:** Cassava (17%), Beans (16%), Bananas (matooke) (15.5%), Maize (14%), Coffee (9.6%) S. Potatoes (7%), Groundnuts (3.8%), Finger millet (2.1%), Sorghum (1.9%)

| **Crop species** | **Freq.** | **Percent** | **Cum.** |
| --- | --- | --- | --- |
| Rice | 63 | 0.75 | 0.75 |
| Maize | 1,181 | 14.01 | 14.76 |
| Finger millet | 175 | 2.08 | 16.83 |
| Sorghum | 162 | 1.92 | 18.76 |
| Beans | 1,353 | 16.05 | 34.81 |
| Field peas | 36 | 0.43 | 35.24 |
| Cowpeas | 16 | 0.19 | 35.43 |
| Pigeon peas | 10 | 0.12 | 35.54 |
| Chickpeas | 15 | 0.18 | 35.72 |
| Groundnuts | 316 | 3.75 | 39.47 |
| Soya beans | 135 | 1.60 | 41.07 |
| Sunflower | 63 | 0.75 | 41.82 |
| Simsim | 124 | 1.47 | 43.29 |
| Cabbage | 11 | 0.13 | 43.42 |
| Tomatoes | 36 | 0.43 | 43.85 |
| Carrots | 1 | 0.01 | 43.86 |
| Onions | 17 | 0.20 | 44.06 |
| Pumpkins | 1 | 0.01 | 44.07 |
| Dodo | 3 | 0.04 | 44.11 |
| Eggplants | 14 | 0.17 | 44.28 |
| Sugarcane | 58 | 0.69 | 44.96 |
| Tobacco | 46 | 0.55 | 45.51 |
| Irish potatoes | 135 | 1.60 | 47.11 |
| Sweet potatoes | 607 | 7.20 | 54.31 |
| Cassava | 1,430 | 16.97 | 71.28 |
| Yam | 33 | 0.39 | 71.67 |
| Cocoyam | 2 | 0.02 | 71.69 |
| Oranges | 16 | 0.19 | 71.88 |
| Pawpaw | 1 | 0.01 | 71.89 |
| Pineapples | 14 | 0.17 | 72.06 |
| Banana food | 1,307 | 15.51 | 87.57 |
| Banana beer | 86 | 1.02 | 88.59 |
| Banana sweet | 67 | 0.79 | 89.38 |
| Mango | 4 | 0.05 | 89.43 |
| Jackfruit | 3 | 0.04 | 89.46 |
| Avocado | 5 | 0.06 | 89.52 |
| Passion fruit | 4 | 0.05 | 89.57 |
| Coffee | 810 | 9.6 | 99.18 |
| Cocoa | 13 | 0.15 | 99.34 |
| Tea | 13 | 0.15 | 99.49 |
| Ginger | 1 | 0.01 | 99.50 |
| Vanilla | 14 | 0.17 | 99.67 |
| Other | 28 | 0.33 | 100.00 |
| Total | 8,429 | 100.00 |  |

**Crops for the Year 2018**:

**Top 9 grown crops:** Beans (16.2%), Maize (16%), Cassava (14.5%), Banana (matooke) (13.6%), Coffee (9.1%) S. Potatoes (8.2%), Sorghum (3.7%), Groundnuts (3%), Finger millet (2.5%)

| **Crop species** | **Freq.** | **Percent** | **Cum.** |
| --- | --- | --- | --- |
| Wheat | 7 | 0.08 | 0.08 |
| Barley | 1 | 0.01 | 0.09 |
| Rice | 71 | 0.81 | 0.90 |
| Maize | 1,404 | 16.00 | 16.90 |
| Finger millet | 221 | 2.52 | 19.42 |
| Sorghum | 322 | 3.67 | 23.09 |
| Beans | 1,424 | 16.23 | 39.31 |
| Field peas | 42 | 0.48 | 39.79 |
| Cowpeas | 19 | 0.22 | 40.01 |
| Pigeon peas | 34 | 0.39 | 40.39 |
| Chickpeas | 9 | 0.10 | 40.50 |
| Groundnuts | 252 | 2.87 | 43.37 |
| Soya beans | 119 | 1.36 | 44.72 |
| Sunflower | 44 | 0.50 | 45.23 |
| Simsim | 150 | 1.71 | 46.93 |
| Cabbage | 12 | 0.14 | 47.07 |
| Tomatoes | 34 | 0.39 | 47.46 |
| Carrots | 1 | 0.01 | 47.47 |
| Onions | 17 | 0.19 | 47.66 |
| Pumpkins | 5 | 0.06 | 47.72 |
| Dodo | 8 | 0.09 | 47.81 |
| Eggplants | 10 | 0.11 | 47.93 |
| Sugarcane | 46 | 0.52 | 48.45 |
| Tobacco | 67 | 0.78 | 49.21 |
| Irish potatoes | 165 | 1.88 | 51.09 |
| Sweet potatoes | 716 | 8.16 | 59.25 |
| Cassava | 1,273 | 14.51 | 73.76 |
| Yam | 30 | 0.34 | 74.10 |
| Cocoyam | 4 | 0.05 | 74.15 |
| Oranges | 17 | 0.19 | 74.34 |
| Pawpaw | 5 | 0.06 | 74.40 |
| Pineapples | 10 | 0.11 | 74.51 |
| Banana food | 1,190 | 13.56 | 88.07 |
| Banana beer | 81 | 0.92 | 88.99 |
| Banana sweet | 77 | 0.88 | 89.87 |
| Mango | 9 | 0.10 | 89.97 |
| Jackfruit | 7 | 0.08 | 90.05 |
| Avocado | 10 | 0.11 | 90.17 |
| Passion fruit | 6 | 0.07 | 90.23 |
| Coffee | 797 | 9.09 | 99.32 |
| Cocoa | 11 | 0.13 | 99.44 |
| Tea | 10 | 0.11 | 99.56 |
| Ginger | 2 | 0.02 | 99.58 |
| Oil palm | 1 | 0.01 | 99.59 |
| Vanilla | 7 | 0.08 | 99.67 |
| Other | 29 | 0.33 | 100.00 |
| Total | 8,776 | 100.00 |  |

**Crops for the Year 2015**:

**Top 9 grown crops:** Cassava (16.7%), Beans (16.6%), Maize (14.6%), Bananas (matooke) (12.8%), S. Potatoes (8%), Coffee (6.7%), Groundnuts (4%), Finger millet (2.5%), Sorghum (2%)

| **Crop species** | **Freq.** | **Percent** | **Cum.** |
| --- | --- | --- | --- |
| Wheat | 4 | 0.04 | 0.04 |
| Barley | 2 | 0.02 | 0.06 |
| Rice | 54 | 0.50 | 0.55 |
| Maize | 1,576 | 14.56 | 15.12 |
| Finger Millet | 275 | 2.54 | 17.66 |
| Sorghum | 256 | 2.37 | 20.02 |
| Beans | 1,799 | 16.62 | 36.64 |
| Field Peas | 47 | 0.43 | 37.08 |
| Cow Peas | 36 | 0.33 | 37.41 |
| Pigeon Peas | 22 | 0.20 | 37.61 |
| Chickpeas | 5 | 0.05 | 37.66 |
| Groundnuts | 443 | 4.09 | 41.75 |
| Soya Beans | 90 | 0.83 | 42.59 |
| Sunflower | 118 | 1.09 | 43.68 |
| Simsim | 305 | 2.82 | 46.49 |
| Cabbage | 19 | 0.18 | 46.67 |
| Tomatoes | 64 | 0.59 | 47.26 |
| Carrots | 1 | 0.01 | 47.27 |
| Onions | 29 | 0.27 | 47.54 |
| Pumpkins | 8 | 0.07 | 47.61 |
| Dodo | 4 | 0.04 | 47.65 |
| Eggplants | 22 | 0.20 | 47.85 |
| Cucumber | 1 | 0.01 | 47.86 |
| Green pepper | 3 | 0.03 | 47.89 |
| Sugarcane | 53 | 0.49 | 48.80 |
| Tobacco | 50 | 0.47 | 48.84 |
| Irish Potatoes | 222 | 2.05 | 50.89 |
| Sweet Potatoes | 873 | 8.07 | 58.96 |
| Cassava | 1,805 | 16.68 | 75.64 |
| Yam | 60 | 0.55 | 76.19 |
| Coco Yam | 4 | 0.04 | 76.23 |
| Oranges | 16 | 0.15 | 76.37 |
| Pawpaw | 10 | 0.09 | 76.47 |
| Pineapples | 13 | 0.12 | 76.59 |
| Banana Food | 1,388 | 12.82 | 89.41 |
| Banana Beer | 90 | 0.83 | 90.24 |
| Banana Sweet | 116 | 1.07 | 91.31 |
| Mango | 41 | 0.38 | 91.69 |
| Jackfruit | 49 | 0.45 | 92.15 |
| Avocado | 73 | 0.67 | 92.82 |
| Passion Fruit | 11 | 0.10 | 92.92 |
| Watermelon | 1 | 0.01 | 92.93 |
| Coffee | 730 | 6.74 | 99.68 |
| Cocoa | 5 | 0.05 | 99.72 |
| Tea | 6 | 0.06 | 99.78 |
| Ginger | 2 | 0.02 | 99.80 |
| Vanilla | 5 | 0.05 | 99.84 |
| Other | 17 | 0.16 | 100.00 |
| Total | 10,823 | 100.00 |  |

**Overall rank of 9 most grown crops:** Beans (16.3%), Cassava (16.1%), Maize (14.9%), Bananas (matooke) (13.9%), Coffee (8.5%), S. Potatoes (7.8%), Groundnuts (3.6%), Sorghum (2.7%) Finger millet (2.4%)

**Animals**

**Top 5 Animal species:** Cattle (30.2%), Goats (29.5%), Chicken (23.3%), Pig (8%), Sheep (5.3%)

| **Animal Species** | **2015** | | **2018** | | **2019** | | **Overall** |
| --- | --- | --- | --- | --- | --- | --- | --- |
|  | Freq. | Percent | Freq. | Percent | Freq. | Percent | **Percent** |
| Cattle | 1,588 | 30.17 | 1,479 | 29.33 | 1,676 | 31.11 | 30.20 |
| Donkeys | 2 | 0.04 | 4 | 0.08 | 2 | 0.04 | 0.05 |
| Goats | 1,507 | 28.63 | 1,522 | 30.18 | 1,604 | 29.77 | 29.53 |
| Sheep | 246 | 4.67 | 283 | 5.61 | 306 | 5.68 | 5.32 |
| Pigs | 423 | 8.04 | 389 | 7.71 | 449 | 8.33 | 8.03 |
| Chicken | 1,241 | 23.58 | 1,225 | 24.29 | 1,189 | 22.07 | 23.31 |
| Ducks | 240 | 4.56 | 126 | 2.50 | 140 | 2.60 | 3.22 |
| Rabbits | 17 | 0.32 | 15 | 0.30 | 22 | 0.41 | 0.34 |
| **Total** | 5,264 | 100.00 | 5,043 | 100.00 | 5,388 | 100.00 | 100.00 |
