# Supplementary material for: Farm production diversity, household dietary diversity, and nutrition: Evidence from Uganda’s national panel survey
Source: PLoS One. 2022 Dec 16;17(12):e0279358. doi: 10.1371/journal.pone.0279358 (PMC9757588; doi:10.1371/journal.pone.0279358)
Supplement: S2 Table — (DOCX) [file pone.0279358.s002.docx]

# S2 Table. Association of farm production diversity (FPD) on Household dietary diversity score (HDDS)

| Models | RE (1) | FE (2) | MK (3) | FE (4) | MK (5) | FE (6) | MK (7) |
| --- | --- | --- | --- | --- | --- | --- | --- |
| Variables | HDDS | HDDS | HDDS | HDDS | HDDS | HDDS | HDDS |
| IHS of FPD (bio index) | 0.183*** | -0.210*** | -0.206*** |  |  |  |  |
|  | (0.032) | (0.062) | (0.056) |  |  |  |  |
| IHS of Animal FPD (bio index) |  |  |  | 0.484*** | 0.483*** | 0.485*** | 0.259*** |
|  |  |  |  | (0.078) | (0.071) | (0.079) | (0.043) |
| IHS of Crop FPD (bio index) |  |  |  | -0.561*** | -0.557*** | -0.643*** | -0.070** |
|  |  |  |  | (0.064) | (0.059) | (0.066) | (0.034) |
| Male head (dummy) |  |  |  |  |  | 0.030 | -0.004 |
|  |  |  |  |  |  | (0.262) | (0.226) |
| Mobile phone use (dummy) |  |  |  |  |  | 0.002 | -0.007 |
|  |  |  |  |  |  | (0.107) | (0.093) |
| Age of head (years) |  |  |  |  |  | -0.021* | -0.007 |
|  |  |  |  |  |  | (0.013) | (0.011) |
| Household size (adult equivalents) |  |  |  |  |  | 0.013 | -0.005 |
|  |  |  |  |  |  | (0.039) | (0.032) |
| Education of head (years) |  |  |  |  |  | -0.039 | -0.054** |
|  |  |  |  |  |  | (0.027) | (0.022) |
| Total assets (million UGX) |  |  |  |  |  | -0.038 | -0.125*** |
|  |  |  |  |  |  | (0.036) | (0.027) |
| Experienced shocks (dummy) |  |  |  |  |  | 0.104 | 0.075 |
|  |  |  |  |  |  | (0.102) | (0.088) |
| Land Size (Acres by GPS) |  |  |  |  |  | -0.009 | -0.025 |
|  |  |  |  |  |  | (0.025) | (0.022) |
| Farming is the main income source (dummy) |  |  |  |  |  | -0.003 | -0.083 |
|  |  |  |  |  |  | (0.111) | (0.096) |
| Year is 2018 | 0.138** | -0.085 | 0.127* | -0.084 | 0.127* | -0.085 | 0.102 |
|  | (0.070) | (0.082) | (0.070) | (0.081) | (0.069) | (0.084) | (0.070) |
| Year is 2019 | 0.196*** | -0.107 | 0.186*** | -0.113 | 0.180** | -0.092 | 0.077 |
|  | (0.071) | (0.083) | (0.071) | (0.082) | (0.070) | (0.083) | (0.070) |
| *Means of covariates* |  |  |  |  |  |  |  |
| IHS of FPD (bio index) |  |  | 0.571*** |  |  |  |  |
|  |  |  | (0.068) |  |  |  |  |
| IHS of Animal FPD (bio index) |  |  |  |  | -0.201** |  |  |
|  |  |  |  |  | (0.085) |  |  |
| IHS of Crop FPD (bio index) |  |  |  |  | 0.792*** |  |  |
|  |  |  |  |  | (0.069) |  |  |
| Male head (dummy) |  |  |  |  |  |  | 0.004 |
|  |  |  |  |  |  |  | (0.236) |
| Mobile phone use (dummy) |  |  |  |  |  |  | 0.272** |
|  |  |  |  |  |  |  | (0.128) |
| Age of head (years) |  |  |  |  |  |  | 0.006 |
|  |  |  |  |  |  |  | (0.011) |
| Household size (adult equivalents) |  |  |  |  |  |  | -0.018 |
|  |  |  |  |  |  |  | (0.036) |
| Education of head (years) |  |  |  |  |  |  | 0.052* |
|  |  |  |  |  |  |  | (0.031) |
| Total assets (million UGX) |  |  |  |  |  |  | 0.307*** |
|  |  |  |  |  |  |  | (0.028) |
| Experienced shocks (dummy) |  |  |  |  |  |  | 0.824*** |
|  |  |  |  |  |  |  | (0.176) |
| Land Size (Acres by GPS) |  |  |  |  |  |  | 0.004 |
|  |  |  |  |  |  |  | (0.035) |
| Farming is the main income source |  |  |  |  |  |  | -0.069 |
|  |  |  |  |  |  |  | (0.125) |
| Constant | 7.028*** | 7.986*** | 6.672*** | 8.007*** | 6.751*** | 9.220*** | 5.218*** |
|  | (0.081) | (0.136) | (0.091) | (0.123) | (0.082) | (0.639) | (0.219) |
| Observations | 6,992 | 6,992 | 6,992 | 6,992 | 6,992 | 6,828 | 6,828 |
| No. of households | 2,838 | 2,838 | 2,838 | 2,838 | 2,838 | 2,804 | 2,804 |
| F value |  | 4.46*** |  | 23.22*** |  | 8.72*** |  |
| Hausman test value |  | 105.61*** |  | 155.98*** |  | 158.75*** |  |
| Wald Chi2 value | 41.28*** |  | 112.39*** |  | 217.64*** |  | 329.68*** |

Standard errors in parentheses; *** p<0.01, ** p<0.05, * p<0.1; IHS is Inverse hyperbolic sine
