# Supplementary material for: Farm production diversity, household dietary diversity, and nutrition: Evidence from Uganda’s national panel survey
Source: PLoS One. 2022 Dec 16;17(12):e0279358. doi: 10.1371/journal.pone.0279358 (PMC9757588; doi:10.1371/journal.pone.0279358)
Supplement: S3 Table — (DOCX) [file pone.0279358.s003.docx]

**S3 Table.** **Association of farm production diversity (FPD) and daily energy intake per adult equivalent (AE)**

| Nutrition indicator | Daily energy intake (kilocalories/AE) | | | |
| --- | --- | --- | --- | --- |
| Models | MK (1) | MK (2) | MK (3) | MK (4) |
| Variables | Total energy | Total energy | Own farm-sourced | Markets source |
| IHS of FPD (bio index) | 125.5*** |  |  |  |
|  | (16.38) |  |  |  |
| IHS of Animal FPD (bio index) |  | -12.36 | 2.781*** | -9.891 |
|  |  | (20.30) | (0.866) | (16.19) |
| IHS of Crop FPD (bio index) |  | 154.2*** | 12.24*** | 56.34*** |
|  |  | (16.19) | (0.689) | (12.87) |
| Male head (dummy) | 78.23 | 81.85 | 4.825 | 30.50 |
|  | (87.51) | (87.50) | (3.813) | (72.91) |
| Mobile phone use (dummy) | 9.054 | 10.34 | 1.212 | 6.538 |
|  | (35.88) | (35.88) | (1.564) | (29.91) |
| Age of head (years) | 1.295 | 1.587 | -0.175 | 0.633 |
|  | (4.186) | (4.186) | (0.182) | (3.489) |
| Household size (adult equivalents) | -98.97*** | -100.4*** | 1.668*** | -63.20*** |
|  | (12.44) | (12.44) | (0.541) | (10.32) |
| Education of head (years) | -17.75** | -18.10** | -0.447 | -13.08* |
|  | (8.764) | (8.762) | (0.381) | (7.285) |
| Total assets (million UGX) | -9.490 | -9.040 | 0.467 | -13.90 |
|  | (10.70) | (10.70) | (0.461) | (8.75) |
| Experienced shocks (dummy) | 11.74 | 7.839 | 1.550 | 5.085 |
|  | (34.10) | (34.11) | (1.486) | (28.41) |
| Land Size (Acres by GPS) | 1.778 | 1.795 | 0.034 | -3.732 |
|  | (8.482) | (8.481) | (0.370) | (7.076) |
| Farming is the main income source (dummy) | 47.36 | 48.82 | -0.659 | 51.55* |
|  | (36.99) | (36.99) | (1.611) | (30.81) |
| Year is 2018 | -476.7*** | -476.1*** | -8.850*** | -334.0*** |
|  | (27.50) | (27.49) | (1.196) | (22.85) |
| Year is 2019 | -630.8*** | -630.0*** | -12.36*** | -441.2*** |
|  | (27.46) | (27.45) | (1.195) | (22.83) |
| *Means of covariates* |  |  |  |  |
| Male head (dummy) | -169.2* | -176.9* | -7.642* | -82.81 |
|  | (95.80) | (95.68) | (4.136) | (78.51) |
| Mobile phone use (dummy) | 239.4*** | 215.8*** | 6.929*** | 133.3*** |
|  | (62.09) | (61.93) | (2.612) | (48.44) |
| Age of head (years) | -2.428 | -2.887 | 0.188 | -2.672 |
|  | (4.336) | (4.334) | (0.188) | (3.590) |
| Household size (adult equivalents) | -8.059 | -1.419 | -2.429*** | -11.05 |
|  | (16.05) | (16.10) | (0.687) | (12.87) |
| Education of head (years) | 14.09 | 15.49 | -2.282*** | 24.33** |
|  | (14.90) | (14.83) | (0.626) | (11.63) |
| Total assets (million UGX) | 112.0*** | 109.0*** | 2.280*** | 79.70*** |
|  | (13.10) | (13.01) | (0.553) | (10.30) |
| Experienced shocks (dummy) | -25.32 | 48.80 | 10.22*** | -13.29 |
|  | (87.08) | (87.77) | (3.695) | (68.36) |
| Land Size (Acres by GPS) | -25.02 | -25.28 | 4.376*** | -47.63*** |
|  | (17.80) | (17.70) | (0.741) | (13.66) |
| Farming is the main income source | -113.4* | -105.5* | 28.42*** | -328.8*** |
|  | (59.36) | (59.13) | (2.502) | (46.55) |
| Constant | 3,017*** | 2,998*** | 4.489 | 2,053*** |
|  | (121.5) | (120.7) | (5.025) | (91.92) |
| Observations | 6,828 | 6,828 | 6,828 | 6,828 |
| No. of households | 2,804 | 2,804 | 2,804 | 2,804 |
| Wald Chi2 value | 986.33*** | 1024.91*** | 1409.65*** | 883.99*** |

Standard errors in parentheses; *** p<0.01, ** p<0.05, * p<0.1; IHS is Inverse hyperbolic sine; UGX is Uganda shillings (1USD = 3,557 USD); GPS is Global positioning system
