# Supplementary material for: Farm production diversity, household dietary diversity, and nutrition: Evidence from Uganda’s national panel survey
Source: PLoS One. 2022 Dec 16;17(12):e0279358. doi: 10.1371/journal.pone.0279358 (PMC9757588; doi:10.1371/journal.pone.0279358)
Supplement: S4 Table — (DOCX) [file pone.0279358.s004.docx]

**S4 Table. Association of farm production diversity (FPD) and daily iron intake per adult equivalent (AE)**

| Nutrition indicator | Daily iron intake (milligrams/AE) | | | |
| --- | --- | --- | --- | --- |
| Models | MK (1) | MK (2) | MK (3) | MK (4) |
| Variables | Total | Total | Own farm-sourced | Markets source |
| IHS of FPD (bio index) | 1.089*** |  |  |  |
|  | (0.156) |  |  |  |
| IHS of Animal FPD (bio index) |  | -0.011 | 0.132** | 0.041 |
|  |  | (0.194) | (0.052) | (0.138) |
| IHS of Crop FPD (bio index) |  | 1.246*** | 0.748*** | 0.222** |
|  |  | (0.154) | (0.042) | (0.110) |
| Male head (dummy) | 0.821 | 0.842 | 0.281 | 0.237 |
|  | (0.837) | (0.837) | (0.233) | (0.612) |
| Mobile phone use (dummy) | 0.094 | 0.104 | 0.089 | 0.009 |
|  | (0.343) | (0.343) | (0.096) | (0.251) |
| Age of head (years) | 0.007 | 0.009 | -0.008 | 0.002 |
|  | (0.040) | (0.040) | (0.011) | (0.029) |
| Household size (adult equivalents) | -0.984*** | -0.995*** | 0.077** | -0.337*** |
|  | (0.119) | (0.119) | (0.033) | (0.087) |
| Education of head (years) | -0.146* | -0.148* | -0.022 | -0.111* |
|  | (0.084) | (0.084) | (0.023) | (0.061) |
| Total assets (million UGX) | -0.103 | -0.100 | 0.017 | -0.126* |
|  | (0.102) | (0.102) | (0.028) | (0.074) |
| Experienced shocks (dummy) | 0.069 | 0.041 | 0.116 | -0.095 |
|  | (0.326) | (0.326) | (0.091) | (0.239) |
| Land Size (Acres by GPS) | 0.039 | 0.039 | 0.002 | -0.035 |
|  | (0.081) | (0.081) | (0.023) | (0.059) |
| Farming is the main income source (dummy) | 0.176 | 0.187 | -0.034 | 0.306 |
|  | (0.354) | (0.354) | (0.098) | (0.259) |
| Year is 2018 | -3.218*** | -3.212*** | -0.456*** | -1.448*** |
|  | (0.263) | (0.263) | (0.073) | (0.192) |
| Year is 2019 | -4.017*** | -4.011*** | -0.722*** | -1.436*** |
|  | (0.263) | (0.262) | (0.073) | (0.192) |
| *Means of covariates* |  |  |  |  |
| Male head (dummy) | -2.028** | -2.079** | -0.507** | -0.723 |
|  | (0.915) | (0.914) | (0.252) | (0.662) |
| Mobile phone use (dummy) | 0.824 | 0.653 | 0.320** | 0.184 |
|  | (0.591) | (0.590) | (0.157) | (0.415) |
| Age of head (years) | -0.011 | -0.014 | 0.009 | -0.019 |
|  | (0.041) | (0.041) | (0.012) | (0.030) |
| Household size (adult equivalents) | -0.057 | -0.007 | -0.152*** | -0.157 |
|  | (0.153) | (0.154) | (0.042) | (0.109) |
| Education of head (years) | -0.061 | -0.053 | -0.133*** | 0.047 |
|  | (0.142) | (0.141) | (0.038) | (0.099) |
| Total assets (million UGX) | 0.886*** | 0.863*** | 0.123*** | 0.603*** |
|  | (0.124) | (0.124) | (0.033) | (0.088) |
| Experienced shocks (dummy) | 0.192 | 0.743 | 0.495** | 0.352 |
|  | (0.828) | (0.837) | (0.222) | (0.587) |
| Land Size (Acres by GPS) | 0.033 | 0.034 | 0.268*** | -0.238** |
|  | (0.169) | (0.169) | (0.045) | (0.118) |
| Farming is the main income source | 0.305 | 0.375 | 1.670*** | -1.850*** |
|  | (0.565) | (0.564) | (0.151) | (0.398) |
| Constant | 17.82*** | 17.74*** | -2.232*** | 7.438*** |
|  | (1.153) | (1.150) | (0.301) | (0.795) |
| Observations | 6,828 | 6,828 | 6,828 | 6,828 |
| No. of households | 2,804 | 2,804 | 2,804 | 2,804 |
| Wald Chi2 value | 581.57*** | 602.69*** | 1298.19*** | 284.01*** |

Standard errors in parentheses, *** p<0.01, ** p<0.05, * p<0.1; IHS is Inverse hyperbolic sine; UGX is Uganda shillings (1USD = 3,557 USD); GPS is Global positioning system
