# Supplementary material for: Farm production diversity, household dietary diversity, and nutrition: Evidence from Uganda’s national panel survey
Source: PLoS One. 2022 Dec 16;17(12):e0279358. doi: 10.1371/journal.pone.0279358 (PMC9757588; doi:10.1371/journal.pone.0279358)
Supplement: S5 Table — (DOCX) [file pone.0279358.s005.docx]

**S 5** **Table. Association of farm production diversity (FPD) and daily zinc intake per adult equivalent (AE)**

| Nutrition indicator | Daily zinc intake (milligrams/AE) | | | |
| --- | --- | --- | --- | --- |
| Models | MK (1) | MK (2) | MK (3) | MK (4) |
| Variables | Total | Total | Own farm-sourced | Markets source |
| IHS of FPD (bio index) | 0.764*** |  |  |  |
|  | (0.115) |  |  |  |
| IHS of Animal FPD (bio index) |  | 0.025 | 0.160*** | 0.032 |
|  |  | (0.143) | (0.047) | (0.102) |
| IHS of Crop FPD (bio index) |  | 0.865*** | 0.663*** | 0.271*** |
|  |  | (0.115) | (0.038) | (0.082) |
| Male head (dummy) | 0.527 | 0.540 | 0.251 | 0.128 |
|  | (0.609) | (0.610) | (0.210) | (0.461) |
| Mobile phone use (dummy) | 0.227 | 0.234 | 0.084 | 0.106 |
|  | (0.250) | (0.250) | (0.086) | (0.189) |
| Age of head (years) | 0.008 | 0.009 | -0.007 | 0.002 |
|  | (0.029) | (0.029) | (0.010) | (0.022) |
| Household size (adult equivalents) | -0.380*** | -0.387*** | 0.060** | -0.212*** |
|  | (0.087) | (0.087) | (0.029) | (0.065) |
| Education of head (years) | -0.109* | -0.111* | -0.019 | -0.073 |
|  | (0.061) | (0.061) | (0.021) | (0.046) |
| Total assets (million UGX) | -0.090 | -0.088 | 0.018 | -0.086 |
|  | (0.075) | (0.075) | (0.025) | (0.055) |
| Experienced shocks (dummy) | 0.049 | 0.031 | 0.099 | -0.015 |
|  | (0.237) | (0.238) | (0.082) | (0.180) |
| Land Size (Acres by GPS) | -0.012 | -0.012 | 0.003 | -0.024 |
|  | (0.059) | (0.059) | (0.020) | (0.045) |
| Farming is the main income source (dummy) | 0.207 | 0.215 | -0.025 | 0.229 |
|  | (0.258) | (0.258) | (0.089) | (0.195) |
| Year is 2018 | -2.485*** | -2.481*** | -0.449*** | -1.503*** |
|  | (0.192) | (0.192) | (0.066) | (0.144) |
| Year is 2019 | -2.785*** | -2.780*** | -0.684*** | -1.396*** |
|  | (0.191) | (0.191) | (0.066) | (0.144) |
| *Means of covariates* |  |  |  |  |
| Male head (dummy) | -1.205* | -1.240* | -0.437* | -0.370 |
|  | (0.671) | (0.671) | (0.228) | (0.497) |
| Mobile phone use (dummy) | 1.014** | 0.896** | 0.314** | 0.482 |
|  | (0.442) | (0.442) | (0.142) | (0.307) |
| Age of head (years) | -0.019 | -0.021 | 0.009 | -0.017 |
|  | (0.030) | (0.030) | (0.010) | (0.023) |
| Household size (adult equivalents) | -0.194* | -0.163 | -0.113*** | -0.158* |
|  | (0.113) | (0.114) | (0.038) | (0.081) |
| Education of head (years) | -0.067 | -0.061 | -0.120*** | 0.029 |
|  | (0.106) | (0.106) | (0.034) | (0.074) |
| Total assets (million UGX) | 0.826*** | 0.810*** | 0.119*** | 0.513*** |
|  | (0.093) | (0.093) | (0.030) | (0.065) |
| Experienced shocks (dummy) | 0.027 | 0.391 | 0.484** | 0.053 |
|  | (0.621) | (0.627) | (0.201) | (0.433) |
| Land Size (Acres by GPS) | 0.008 | 0.007 | 0.223*** | -0.198** |
|  | (0.127) | (0.127) | (0.040) | (0.087) |
| Farming is the main income source | -0.094 | -0.053 | 1.532*** | -1.383*** |
|  | (0.422) | (0.421) | (0.136) | (0.295) |
| Constant | 7.817*** | 7.773*** | -2.898*** | 3.862*** |
|  | (0.871) | (0.868) | (0.271) | (0.583) |
| Observations | 6,828 | 6,828 | 6,828 | 6,828 |
| No. of households | 2,804 | 2,804 | 2,804 | 2,804 |
| Wald Chis2 value | 529.53*** | 548.02*** | 1353.46*** | 396.52*** |

Standard errors in parentheses; *** p<0.01, ** p<0.05, * p<0.1; IHS is Inverse hyperbolic sine
