# Supplementary material for: Farm production diversity, household dietary diversity, and nutrition: Evidence from Uganda’s national panel survey
Source: PLoS One. 2022 Dec 16;17(12):e0279358. doi: 10.1371/journal.pone.0279358 (PMC9757588; doi:10.1371/journal.pone.0279358)
Supplement: S6 Table — (DOCX) [file pone.0279358.s006.docx]

**S6 Table. Association of farm production diversity (FPD) and daily vitamin-A intake per adult equivalent (AE)**

| Nutrition indicator | Daily vitamin A intake (rae_micrograms/AE) | | | |
| --- | --- | --- | --- | --- |
| Models | MK (1) | MK (2) | MK (3) | MK (4) |
| Variables | Total | Total | Own farm-sourced | Markets source |
| IHS of FPD (bio index) | 18.72*** |  |  |  |
|  | (3.988) |  |  |  |
| IHS of Animal FPD (bio index) |  | 16.75*** | 0.556*** | 4.483*** |
|  |  | (4.950) | (0.188) | (1.342) |
| IHS of Crop FPD (bio index) |  | 12.66*** | 2.324*** | -1.030 |
|  |  | (3.945) | (0.149) | (1.067) |
| Male head (dummy) | 40.17* | 39.54* | 0.659 | 0.796 |
|  | (21.48) | (21.51) | (0.865) | (6.114) |
| Mobile phone use (dummy) | 0.670 | 0.957 | 0.323 | -4.284* |
|  | (8.809) | (8.820) | (0.355) | (2.508) |
| Age of head (years) | 0.098 | 0.048 | -0.056 | 0.234 |
|  | (1.028) | (1.029) | (0.041) | (0.293) |
| Household size (adult equivalents) | -7.306** | -7.189** | 0.428*** | -0.698 |
|  | (3.051) | (3.055) | (0.122) | (0.864) |
| Education of head (years) | -3.170 | -3.232 | -0.091 | 0.081 |
|  | (2.150) | (2.153) | (0.086) | (0.611) |
| Total assets (million UGX) | -2.810 | -3.020 | 0.102 | -1.650** |
|  | (2.610) | (2.620) | (0.103) | (0.731) |
| Experienced shocks (dummy) | 3.455 | 3.850 | 0.488 | -0.264 |
|  | (8.370) | (8.383) | (0.337) | (2.382) |
| Land Size (Acres by GPS) | -3.774* | -3.775* | -0.131 | -1.144* |
|  | (2.083) | (2.085) | (0.084) | (0.594) |
| Farming is the main income source (dummy) | 2.752 | 2.676 | -0.034 | 1.286 |
|  | (9.081) | (9.091) | (0.366) | (2.584) |
| Year is 2018 | -61.96*** | -61.98*** | -1.088*** | -14.19*** |
|  | (6.747) | (6.754) | (0.271) | (1.915) |
| Year is 2019 | -51.02*** | -51.05*** | -1.412*** | -10.92*** |
|  | (6.738) | (6.745) | (0.271) | (1.914) |
| *Means of covariates* |  |  |  |  |
| Male head (dummy) | -61.11*** | -60.90*** | -1.068 | -1.694 |
|  | (23.43) | (23.44) | (0.925) | (6.562) |
| Mobile phone use (dummy) | -38.80*** | -38.78*** | 0.881 | -4.505 |
|  | (15.03) | (15.03) | (0.558) | (4.003) |
| Age of head (years) | -0.370 | -0.324 | 0.086** | -0.532* |
|  | (1.063) | (1.064) | (0.043) | (0.301) |
| Household size (adult equivalents) | -2.640 | -3.426 | -0.401*** | -2.827*** |
|  | (3.903) | (3.924) | (0.150) | (1.069) |
| Education of head (years) | -1.800 | -1.807 | -0.237* | -0.232 |
|  | (3.607) | (3.601) | (0.134) | (0.961) |
| Total assets (million UGX) | 10.40*** | 10.40*** | 0.310*** | 3.17*** |
|  | (3.17) | (3.17) | (0.120) | (0.855) |
| Experienced shocks (dummy) | 63.82*** | 60.89*** | 1.123 | 11.78** |
|  | (21.05) | (21.29) | (0.785) | (5.641) |
| Land Size (Acres by GPS) | 10.72** | 10.66** | 1.074*** | -2.016* |
|  | (4.296) | (4.285) | (0.156) | (1.125) |
| Farming is the main income source | 49.73*** | 48.36*** | 4.635*** | -13.71*** |
|  | (14.39) | (14.37) | (0.538) | (3.852) |
| Constant | 524.0*** | 532.0*** | -3.694*** | 101.9*** |
|  | (29.23) | (29.16) | (1.044) | (7.540) |
| Observations | 6,828 | 6,828 | 6,828 | 6,828 |
| No. of households | 2,804 | 2,804 | 2,804 | 2,804 |
| Wald Chi2 value | 240.75*** | 247.29*** | 1062.00*** | 212.63*** |

Standard errors in parentheses; *** p<0.01, ** p<0.05, * p<0.1; IHS is Inverse hyperbolic sine
