# Supplementary material for: Farm production diversity, household dietary diversity, and nutrition: Evidence from Uganda’s national panel survey
Source: PLoS One. 2022 Dec 16;17(12):e0279358. doi: 10.1371/journal.pone.0279358 (PMC9757588; doi:10.1371/journal.pone.0279358)

I contacted Dr. Gerd Reucker for the permissions as in the email screen shot below, but he too didn't have the original information needed to grant permissions on the map. See in screen shot below

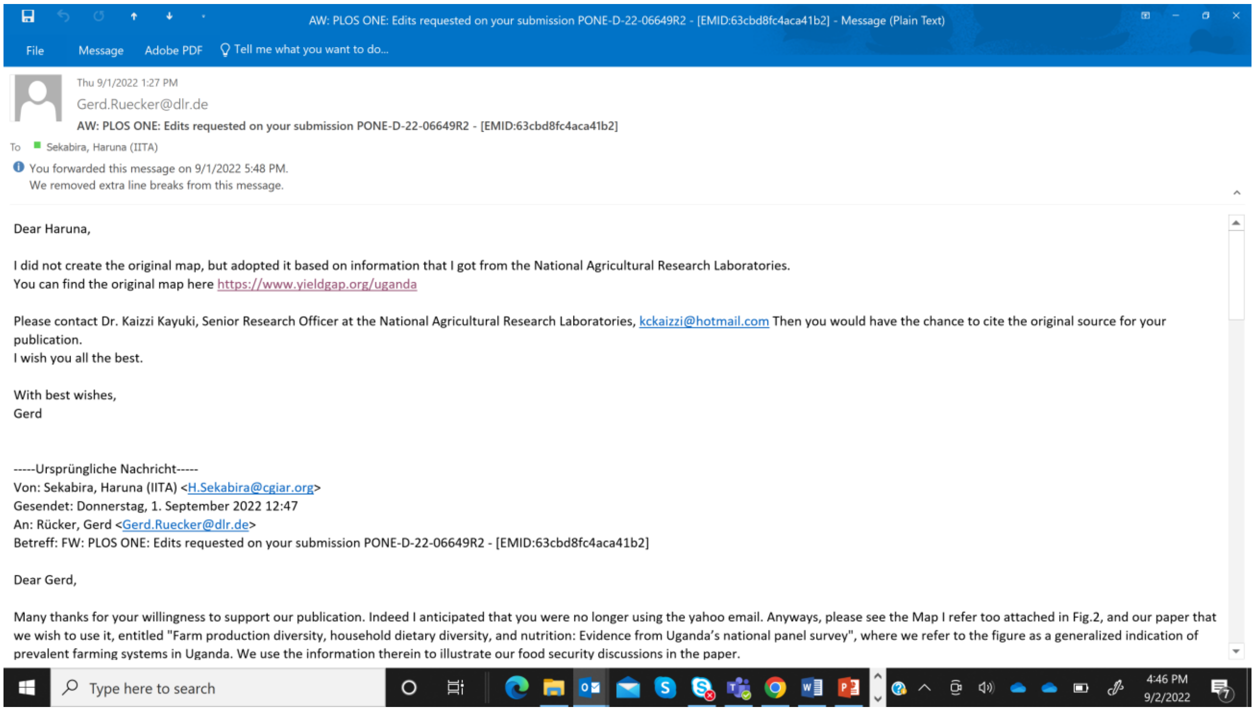

AW: PLOS ONE: Edits requested on your submission PONE-D-22-06649R2 - [EMID:63cbd8fc4aca41b2] - Message (Plain Text)

File Message Adobe PDF Tell me what you want to do...

Thu 9/1/2022 1:27 PM
Gerd.Ruecker@dlr.de
AW: PLOS ONE: Edits requested on your submission PONE-D-22-06649R2 - [EMID:63cbd8fc4aca41b2]
To Sekabira, Haruna (IITA)
You forwarded this message on 9/1/2022 5:48 PM.
We removed extra line breaks from this message.

Dear Haruna,

I did not create the original map, but adopted it based on information that I got from the National Agricultural Research Laboratories.
You can find the original map here https://www.yieldgap.org/uganda

Please contact Dr. Kaizzi Kayuki, Senior Research Officer at the National Agricultural Research Laboratories, kckaizzi@hotmail.com Then you would have the chance to cite the original source for your publication.
I wish you all the best.

With best wishes,
Gerd

-----Ursprüngliche Nachricht-----
Von: Sekabira, Haruna (IITA) <H.Sekabira@cgiar.org>
Gesendet: Donnerstag, 1. September 2022 12:47
An: Rücker, Gerd <Gerd.Ruecker@dlr.de>
Betreff: FW: PLOS ONE: Edits requested on your submission PONE-D-22-06649R2 - [EMID:63cbd8fc4aca41b2]

Dear Gerd,

Many thanks for your willingness to support our publication. Indeed I anticipated that you were no longer using the yahoo email. Anyways, please see the Map I refer too attached in Fig.2, and our paper that we wish to use it, entitled "Farm production diversity, household dietary diversity, and nutrition: Evidence from Uganda's national panel survey", where we refer to the figure as a generalized indication of prevalent farming systems in Uganda. We use the information therein to illustrate our food security discussions in the paper.

Type here to search
4:46 PM
9/2/2022

On advice from Gerd that he didn't have the original files, I tried to contact Dr. Kayizzi as below

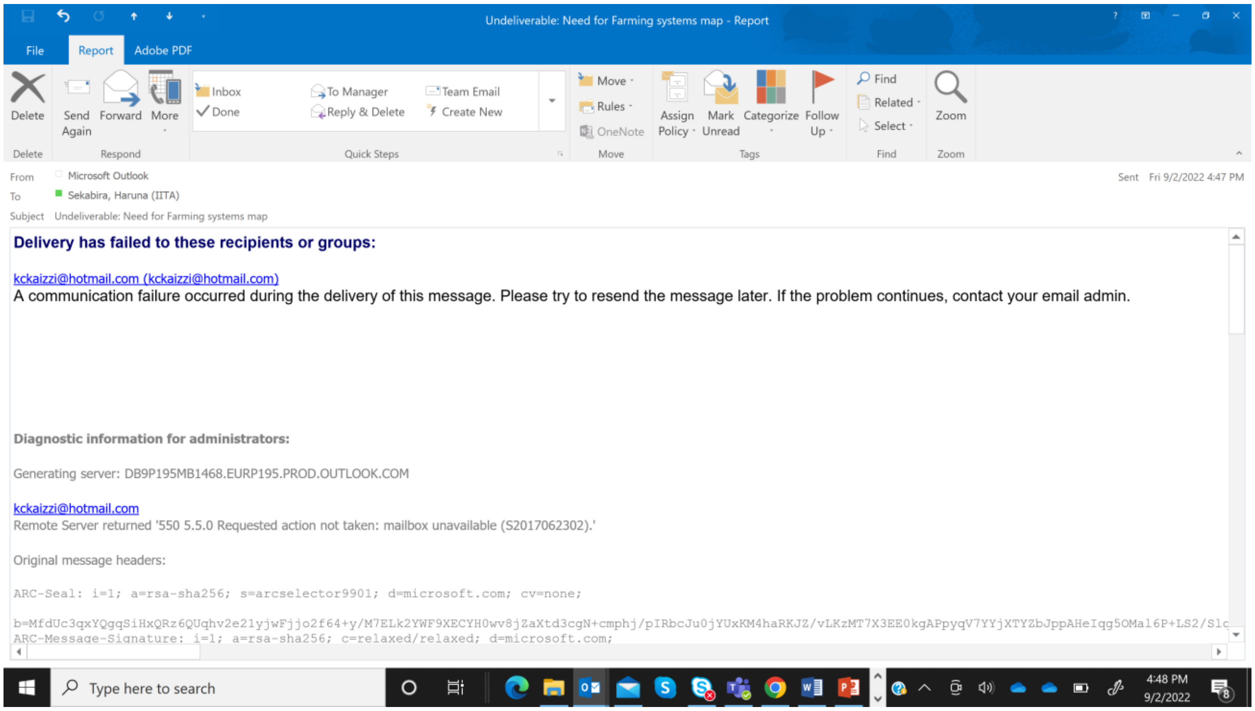

Undeliverable: Need for Farming systems map - Report

File Report Adobe PDF

Delete | Send Again | Forward | More | Inbox | Done | To Manager | Reply & Delete | Team Email | Create New | Move | Rules | OneNote | Assign Policy | Mark Unread | Categorize | Follow Up | Find | Related | Select | Zoom

From Microsoft Outlook
To Sekabira, Haruna (IITA)
Subject Undeliverable: Need for Farming systems map
Sent Fri 9/2/2022 4:47 PM

Delivery has failed to these recipients or groups:

kckaizzi@hotmail.com (kckaizzi@hotmail.com)
A communication failure occurred during the delivery of this message. Please try to resend the message later. If the problem continues, contact your email admin.

Diagnostic information for administrators:

Generating server: DB9P195MB1468.EURP195.PROD.OUTLOOK.COM

kckaizzi@hotmail.com
Remote Server returned '550 5.5.0 Requested action not taken: mailbox unavailable (S2017062302).'

Original message headers:

ARC-Seal: i=1; a=rsa-sha256; s=arcselector9901; d=microsoft.com; cv=none;
b=MfdUc3qxYQgqSiHxQRz6QUqhv2e21yjwFjjo2f64+y/M7ELk2YWF9XECYH0wv8jZaXtd3cgN+cmphj/pIRbcJu0jYUxKM4haRKJZ/vLKzMT7X3EE0kgAPpyqV7YYjXTYZbJppAHeIqg5OMal6P+LS2/Slc
ARC-Message-Signature: i=1; a=rsa-sha256; c=relaxed/relaxed; d=microsoft.com;

Type here to search
4:48 PM
9/2/2022

Because Dr. Kayizzi's email was no longer functional, I went to the Global yield Gap Atlas website as directed by Gerd, where I found in the image below that actually their data is available for use under the CC 4.0 license; see the quotation on the right-hand side of the screen shot

https://www.yieldgap.org/uganda#

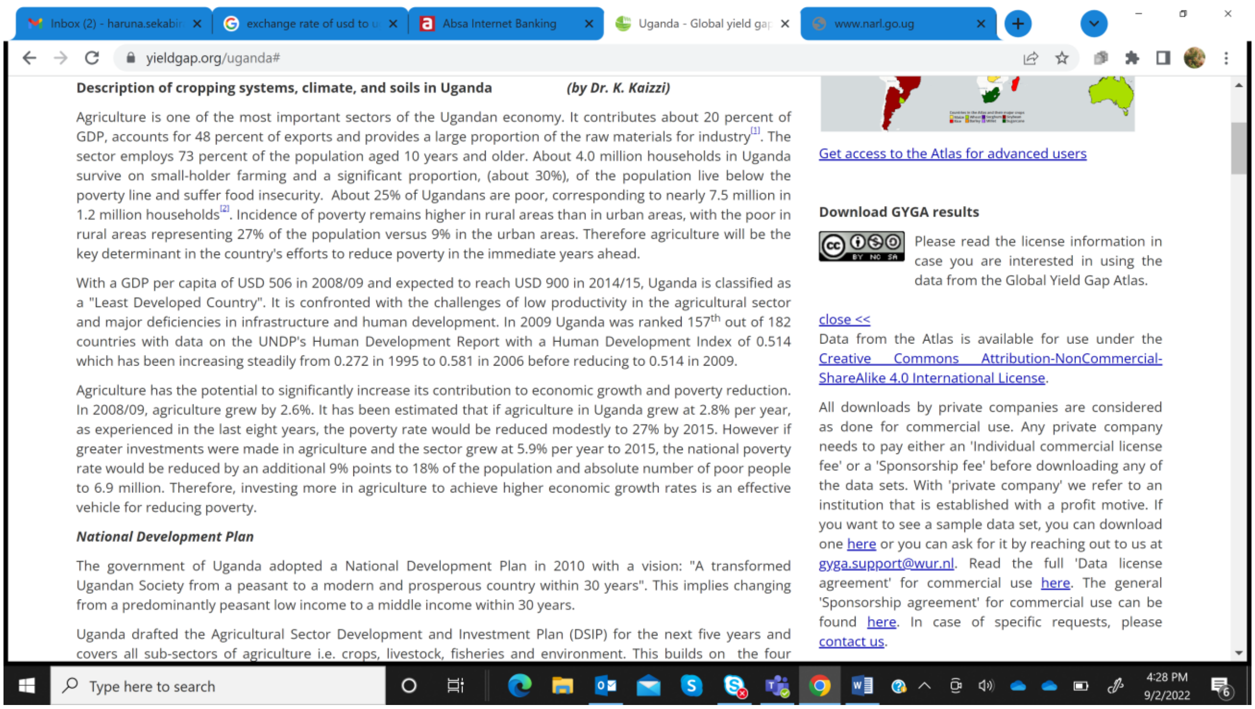

Then I changed the image cited from Gerd Reucker et al to one directly accessed from the Global Yield Gap atlas as seen below – which I cite in the paper as the reference below.

Global Yield Gap Atlas (2022). Description of cropping systems, climate, and soils in Uganda. Accessed from: https://www.yieldgap.org/uganda# on 2nd September 2022

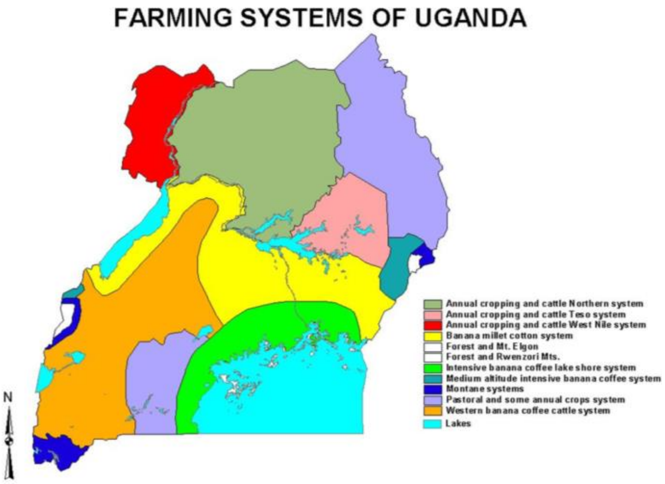

Supplement: Attachment — Submitted filename: Response to Editors for CC 4_0 licence.pdf [file pone.0279358.s011.pdf]
