# Supplementary material for: Farm production diversity, household dietary diversity, and nutrition: Evidence from Uganda’s national panel survey
Source: PLoS One. 2022 Dec 16;17(12):e0279358. doi: 10.1371/journal.pone.0279358 (PMC9757588; doi:10.1371/journal.pone.0279358)
Supplement: Attachment — Submitted filename: Response to Reviewers.pdf [file pone.0279358.s012.pdf]

PONE-D-22-06649R2
Farm Production Diversity, Household Dietary Diversity and Nutrition: Evidence from Uganda's National Panel Survey
PLOS ONE

Our responses to the editor and reviewers' comments/suggestions are marked in red

Editors' comments

There are still some minor issues to solve: the main one regards the functional specification used in the estimates. As one of the reviewers indicated, you should include only the IHS transforms of crop and animal diversity without the linear term. Please, calculate also the marginal effects from parameter estimates following the formulas provided by Bellemare (Oxford Bulletin of Economics and Statistics). This is noted and has been extensively worked upon, as exactly the reviewer guided.

Please submit your revised manuscript by Dec 10 2022 11:59 PM. If you will need more time than this to complete your revisions, please reply to this message or contact the journal office at plosone@plos.org. When you're ready to submit your revision, log on to https://www.editorialmanager.com/pone/ and select the 'Submissions Needing Revision' folder to locate your manuscript file. resubmission of the revised manuscript has been made within the 10th of December deadline.

Please include the following items when submitting your revised manuscript:
•A rebuttal letter that responds to each point raised by the academic editor and reviewer(s). You should upload this letter as a separate file labeled **'Response to Reviewers'**. This file has been constructed and uploaded in the revisions.
•A marked-up copy of your manuscript that highlights changes made to the original version. You should upload this as a separate file labeled **'Revised Manuscript with Track Changes'**. This file has been constructed and uploaded in the revisions.
•An unmarked version of your revised paper without tracked changes. You should upload this as a separate file labeled **'Manuscript'**. This file has been constructed and uploaded in the revisions.

If you would like to make changes to your financial disclosure, please include your updated statement in your cover letter. Guidelines for resubmitting your figure files are available below the reviewer comments at the end of this letter. No changes made.

Please review your reference list to ensure that it is complete and correct. If you have cited papers that have been retracted, please include the rationale for doing so in the manuscript text or remove these references and replace them with relevant current references. **Any changes to the reference list should be mentioned in the rebuttal letter that accompanies your revised manuscript.** If you need to cite a retracted article, indicate the article's retracted status in the References list and also include a citation and full reference for the retraction notice. Two references of Bellemare and Wichman (2020) and Bellemare et al. (2013) – with empirical guidance on IHS implementation and usage have been added.

Reviewers' comments:

Reviewer #1:

As noted in an earlier report, this paper examines associations between the diversity of crop and livestock products produced by households living in Uganda and measures of the diversity of their food consumption. This is a welcome addition to the literature as it includes new evidence from a country that previously had received relatively little attention. And again, the authors are to be commended for taking seriously the concerns raised in my second report and for extensively revising the paper. We are delighted with the observation and comment from the reviewer.

Unfortunately, however, they have misunderstood a crucial comment that I had made. The issue is this. They seek to look at the associations between crop and animal diversity in production and measures of food consumption and food consumption diversity. They want to allow this relationship to be non-linear. However, they cannot use a quadratic specification because the second order term may have a negative coefficient, implying that at some point increased production diversity lowers consumption diversity. They cannot use a logarithmic specification because they may have zero values for their measures of production diversity. This guidance is appreciated and plausible and has been incorporated as earlier advised by the reviewer.

The solution to this problem is to use an inverse hyperbolic sine (see Bellemare, Oxford Bulletin of Economics and Statistics); this allows for increases at a diminishing rate while also allowing for zero values in the independent variable (note that you will need to convert the parameter estimates to marginal effects; Bellemare provides the formulae for doing so). The authors include this in their model but also include a linear term. It does not make sense to include both. The models reported in Tables 2-6 should only include the IHS transforms of crop and animal diversity as well as other control variables. This guiding solution has now been extensively implemented as guided by the reviewer, following Bellemare and Wichman 2020, hopefully to the satisfaction of the reviewer.

Reviewer #2:
(No Response) This is noted, and we are delighted to have satisfied the reviewer.

Reviewer #3:
1. Justifications for indicators selection (lines 95 – 160) are a little too long-winded. Although some redundancy can be useful, this section would be greatly improved by making it a lot more concise. Guidance is noted, and the section is shortened more concisely.

2. Re: "The reviewer makes a very valid observation that indeed the population consumes fewer protein foods as would be expected of rural populations, and we agree that this is noble to study. Unfortunately, we are building on our 2021 work where in the original data, we did not compute for proteins. However, we make a stark recommendation to expand consideration of macro and micronutrient studies to other nutrients, even though such wider coverage may be difficult in one paper, but done individually for each macro or micronutrient. The other constraint is that we may not cover every macro and micronutrient

study in one paper, but we try our best to give a fair picture that can be compared across micronutrients, without actually getting lost into so much detail."

Yes, it would be useful to acknowledge the shortcoming and why you thought studying protein (and other micronutrients) in future research is important. But I don't seem to be able to locate these in the manuscript. Indeed, as observed by the reviewer, this was not included in the manuscript, but we now concisely make mention of it. We are grateful for this advice.
