# Supplementary material for: Farm production diversity, household dietary diversity, and nutrition: Evidence from Uganda’s national panel survey
Source: PLoS One. 2022 Dec 16;17(12):e0279358. doi: 10.1371/journal.pone.0279358 (PMC9757588; doi:10.1371/journal.pone.0279358)
Supplement: Attachment — Submitted filename: Response to Reviewers.docx [file pone.0279358.s009.docx]

**Editors:**

The title page and the general manuscript have been reshaped based on the templates from PLOS ONE. Funding information has also been clearly indicated in the cover letter.

**Reviewer #1:** Review of: Farm Production Diversity, Household Dietary Diversity and Nutrition: Evidence from Uganda’s National Panel Survey (Manuscript Number: PONE-D-22-06649). This paper examines associations between the diversity of crop and livestock products produced by households living in Uganda and measures of the diversity of their food consumption. Although there are already many papers published on this topic, this is a welcome addition to the literature as it includes new evidence from a country that previously had received relatively little attention. That said, the paper would benefit from revisions along a number of lines.

(1) The discussion section needs to be more clear as to how the results presented in this paper fit into the literature. Specifically, I would strongly encourage the authors to place their paper within the debate found in a comparison of the review papers by Jones, A.D., 2017. (Critical review of the emerging research evidence on agricultural biodiversity, diet diversity, and nutritional status in low- and middle-income countries. Nutrition. Reviews. 75, 769–782) and Sibhatu, K. T., & Qaim, M. 2018. (Review: Meta-analysis of the association between production diversity, diets and nutrition in smallholder farm households. Food Policy, 77, 1–18.) Both papers provide empirical reviews on the links between production diversity and diversity of food consumption but reach different conclusions. Jones sees a “strong” link between production and consumption diversity; by contrast, Sibhatu and Qaim argue that “The average marginal effect of production diversity on dietary diversity is positive but small”. This comparison is clearly now done but placing our paper well in-between these two papers – that indeed we find strong (significant) associations between farm production diversity and nutrition outcomes, but the proportional magnitudes of these increments are small – and mostly realized from the diversity in crops species.
My sense is that the paper’s findings are more consistent with the view put forward by Sibhatu and Qaim. Put differently, the authors do not give sufficient attention to the magnitudes of the associations that they observe in their data, both in terms of contextualizing their findings and also in their description of the policy implications. If indeed the magnitudes are small, then it is not obvious that so much attention should be paid to encouraging production diversification. Sometimes, small magnitudes can be substantial proportionally and where we achieve these, especially with crop species for instance with iron 3.4% and zinc 6% proportional increments – indeed it becomes plausible to encourage diversification in such sources.

(2) It is not entirely clear if the sample includes both urban and rural households. This is not stated in the description of the survey, though a urban dummy variable appears in the reported regression results. We now clearly state that the sample is rural and we leave out all urban households. **My strong suggestion would be to focus solely on rural households**. This guidance is effected. Urban households generally grown little food of their own and my guess is that those who do tend to be less well-off (which may explain the odd results the authors sometimes get on the crop diversity variable).

(3) **Regional dummy variables appear in the regression results**; it is not clear why they are being reported when the estimator is a household fixed effect specification (which should difference out time invariant characteristics such as location). Regional dummies are removed and FE re-estimated.

(4) The description of the survey suggests that data are collected twice per year. It is not clear how this is accounted for in the model specification. We clearly state that data is collected once a year even though the naming of the waves, coincided with the planning years of UBOS, an entity which collects the data

(5) Does the measure of crop diversity include non-edible crops such as coffee and cotton? Given the focus of this paper, crop diversity should only include edible crops. Because we have a market component/consumption pathway, aided by sales of both edible and non-edible crops, we included only coffee – which is also sometimes roasted and eaten in households. Essentially, we include only edible crops.

(6) Focusing on Table 2, how much does HDDS vary over time for any given household. Particularly with a fixed effect specification, I wonder if one of the reasons why the parameter estimates are so small is because for many households, there is no change in HDDS over time. A similar concern applies to the index variables for FPD, crops and animals**.** We add a figure to show the variation of HDDS – and further yearly values depicting variations are elaborated in Table 1. It is also made clear that data was collected once every year.

(7) A strength of the paper is that it pays attention to the potentially confounding role of time invariant effects, specifically through the use of random effects, fixed effects and the Mundlak pseudo-fixed effects estimators. But strikingly, all three produce very similar parameter estimates. Given this, and given that the test statistics consistently report rejecting random effects in favor of a fixed effects specification, I suggest dropping the random effects results from the paper and retaining the results from the other two estimation methods. This is perfectly considered and has reduced the columns in Table 2, to a sizeable 6. For Table 2, this change will create some space that can be used to present additional results. I suggest that the authors show the following:

Column 1: FE model with FPD bio index, no other controls
Column 2: MK model with FPD bio index, no other controls
Column 3: FE model with animal bio index & crop bio index, no other controls
Column 4: MK model with animal bio index & crop bio index, no other controls
Column 5: FE model with animal bio index & crop bio index, full set of controls
Column 6: MK model with animal bio index & crop bio index, full set of controls
This set of specifications will allow your readers to see that the results are not sensitive to either the use of FE or MK, or to the inclusion/exclusion of other control variables This has been perfectly adapted, and indeed as the reviewers advised this is now well displayed by our results that they are consistent despite inclusions or exclusions of other controls

(8) I suggest that the authors break Table 3 into four parts (one for each micro-nutrient). For each micro-nutrient, I suggest that they present four results:
Column 1: MK model. Total intake (=Own farm + market) with FPD bio index, full set of controls
Column 2: MK model. Total intake (=Own farm + market) with animal bio index & crop bio index, full set of controls
Column 3: MK model. Own farm intake with animal bio index & crop bio index, full set of controls
Column 4: MK model. Market intake with animal bio index & crop bio index, full set of controls
This reporting would be more informative than what currently exists in the paper. This is perfectly considered, and now each micronutrient is presented in own Table. It would allow you to see the total effect of production diversity on micro-nutrient consumption (column 1); how much of this effect comes from animals and how much from crops (column 2); and whether own production is crowding out market intake (by comparing results from columns 3 and 4). This is now achieved and various impact of FPD or its components is now well documented and the crowding effects also clearly elaborated.

**Reviewer #2:** This is a relevant manuscript that highlights the importance of producing diverse food products. My comments for consideration are below:
• More information is needed in the introduction section describing the common food production and consumption patterns in Uganda. This is now sufficiently done. What various types of crops and livestock are usually produced? This is also done. This is important because HDD score depends on food groups. Also, what proportion of farmers eat their own farm produce and what proportion do not. This is done. In other words, what proportion of own production is consumed? What types of farm products are usually sold for cash and not consumed by farmers? This is also sufficiently done.
• A few specific examples of crops considered in the counts would be useful since nutrient content and expected nutritional contributions may differ among crops. This is also done. Such information will be helpful in the discussions on page 17.
• The authors found a strong positive association of the crop species count with energy and micronutrients sourced from own farm produce and attributed this to farmers mostly consuming what they grow. So, my question is: what is the problem in Uganda? Farm production is still dominantly subsistence and perhaps insufficient to cater for all households’ nutritional and dietary needs. If farmers are producing and eating what they grow, then what is the gap? Why are they hungry or suffering from malnutrition? Largely grow cereals and tubers that mostly supply energy with minimal micronutrient contributions, and often times are produced in insufficient quantities that can’t fully satisfy all food and dietary needs of households.
• In the last sentence on Page 18, what do you mean by essential foods? You listed cereals or their products, oils and fats etc. Why do you refer to these foods as essential? The word “essential” was perhaps used in a wrong context, this is clarified with better wording. Also, you need to distinguish between raw produce and processed products. Aren't the farmers producing cereals and foods rich in fats and oils e.g. groundnuts? Clarify this essential food category. This is well clarified now.
• You concluded that diversification in crop species could be more important than animal species diversification towards availing more energy, and micronutrients per adult. Were you expecting significant differences in nutritional contrition of different livestock species? What different species were counted? Unfortunately, we didn’t have fish production – but indeed the expectation would be valid that different animal species could contribute differently, just as is with crop species, say cereals versus pulses. Unfortunately, we don’t extend our analysis to specific species assessment for impact on specific nutrition outcomes. Perhaps, livestock and fisheries could have some differences in nutritional value and contributions to HDDS. If there is data on fish production and consumption, then it will be interesting and relevant to consider it in the analysis. we didn’t have data on fish production, and perhaps it happens only with large scale urban farmers who are missed in the UNPS general survey.
• Other comments: the manuscript needs to be better structured with introduction, methods and data analysis sections clearly separated. Last paragraph on page 4 – 5 could be moved to methods section. Information under your current methods section could be put under data analysis. This suggested re-arrangement of sections for a more logical flow, has been incorporated.

**Reviewer #3:** The manuscript addresses an area of great importance to the success of policies, especially in resource-scarce settings. The manuscript is well-organised and easy to follow. I have read the manuscript and have made the following observations which I think the authors need to address before publication can be considered:

1. The manuscript requires significant proofreading, especially checking of punctuation. This has been done effectively, with more short sentences and single punctuation per sentence.

2. Section I: Introduction

a) Please justify why the study only investigated the impact of FPD on daily energy, iron, zinc, and vitamin A, but not any other nutrients. Particularly, how do these relate to the nutritional issues in Uganda? Selected micronutrients are justified in the introduction.

3. Section C: Data Description

a) It would be helpful for reader to give an overview of farm characteristics of the sample, e.g., location on map, location map is difficult because it considers all Uganda, so we feared it could add no value. names and types of crops cultivated this is clearly added, cropping system etc.

b) Referring to Table 1, the amount of energy and most micronutrients consumed from both market and own farm sources are far higher in year 2015 than in 2018 and 2019. Is there any explanation to this anomaly? This is observed but clear explanation a bit difficult to zero down too. Perhaps, could it have been due to larger land size and increasing land fragmentation in later years? We Checked-out rains data for 2015 versus other years and other specialties that could have favored more production, but this is available on regional level unlike particular locations for smallholder farmers locations. Could this anomaly affect the statistical rigour?

4. Methods, Results and Discussions

a) It appears that the volume of crop yield and its potential confounding effect on HDDS and nutritional outcome have not been considered or discussed sufficiently in the manuscript. Volume of crop yield couldn’t be considered in the model for fear of potential endogeneity with other X variables including FPD itself. Moreover, our outcome indicator – HDDS - is more of a quality indicator than a quantity one. Nevertheless, we acknowledge sufficiently now in our discussions that a limited volume of crop yield over the years could explain the minimal variation in nutrition outcomes over the years.

b) “Each crop species added to those farmed with in a household was associated with 5.9 and 18.8 kilocalories (0.6 and 0.9 percentage points) added to energy sourced from own farm produce consumption or markets respectively. With regards to iron, each additional crop species was associated with 0.4 and 0.1 milligrams (4.2 and 0.7 percentage points) added to daily iron intake sourced from own farm produce or markets consumption respectively. With regards to zinc, each additional crop species grown on the farm was associated with 0.3 and 0.1 milligrams (6.2 and 0.9 percentage points) added to daily zinc intake sourced from own farm produce, and markets consumption respectively. Lastly, each additional crop species grown on farm was associated with 1.1 and 0.6 rae_micrograms (0.3 and 0.2 percentage points) added to daily vitamin A intake sourced from own farm produce, and markets consumption respectively.”

What is the significance of the percentages of gains quoted above in the diets of the sample population? Are these considered meaningful impact on nutritional status? Also, will increment in crop diversity continue to produce nutritional gains at a linear rate or will the effect level off? At what point will the effect level off? **We added squared FPD to detect linear contributions, which are of course not continuous indefinitely, we discuss this in the paper.** Check for the meaningful impact of changes by % in nutrition status. Percentages are just used to show relative proportions – however, we quote the exact thresholds for each micronutrient as advised by WHO and FAO, the percentages discussed in the paper, just help us to show the proportion of the effect, with regards to average values in the sample; which was earlier compared with thresholds to define energy and micronutrient deficient households and those that were not deficient.

c) “There are however other factors that are consistently and significantly associated with energy, and micronutrients intake, for instance gender effects, household size, and year variables, which we don’t discuss here to prioritize our focus on FPD sub-components, which are our main covariates.”

Wouldn’t understanding these other factors that are also consistently and significantly associated with energy, and micronutrients intake and the relationships of these factors with FPD help in devising more effective policy? Because we increased the coverage of the micronutrients and energy, accommodating each to own table, thus generating 4 tables from 1 original table – still we found ourselves limited by space. Therefore, we completely eliminated all other covariates from the main manuscript and thus their discussions but presented full tables in supporting information in Tables 3-6.

5. Section VI: Policy Implications

a) This section is not sufficiently discussed. It is suggested that the government should devote more attention to crop species, but it does not recommend any specific measures. The scope of our analysis didn’t allow us to make specific crop recommendations as our analysis stopped on crops in general without specifications. The authors should focus on how to translate their research into practice at varying levels of scale, considering all other confounders and trade-offs. We clarify that finer guidance may need further research on particular crops or animal species that we didn’t consider in this study.

b) For various apparent reasons, the policymakers might be more inclined to invest in promotion of cash crops as a means to achieve rural development and poverty alleviation. Cash crops also encourages mono-cropping that reduces FPD. How can this dilemma be addressed? How can policy makers be convinced? Again, our focus didn’t disintegrate particular crops or animals – since there were already two levels of disintegration (at FPD level (animals or crops), and sources of considered nutrition outcomes (own farm or markets), thus further disintegration could be so much in one paper. But we refer to this as an interesting area of research that can be exploited in the future.
