# Supplementary material for: Farm production diversity, household dietary diversity, and nutrition: Evidence from Uganda’s national panel survey
Source: PLoS One. 2022 Dec 16;17(12):e0279358. doi: 10.1371/journal.pone.0279358 (PMC9757588; doi:10.1371/journal.pone.0279358)
Supplement: Attachment — Submitted filename: Review_v2.docx [file pone.0279358.s010.docx]

I find the revision did not fully address my questions or answer.

2. Section I: Introduction

a) Please justify why the study only investigated the impact of FPD on daily energy, iron,

zinc, and vitamin A, but not any other nutrients. Particularly, how do these relate to the

nutritional issues in Uganda? Selected micronutrients are justified in the introduction.

The revised manuscript failed to answer why only energy, iron, zinc and vitamin A are selected. For instance:

- Energy intake does not appear to be a problem in the population, so why are you investigating the impact of FPD on daily energy? Even your results show that “*Average consumption of energy (2,430 kilocalories) was slightly above FAO recommended thresholds per adult*”.
- As a side note, it is often more important to balance the macronutrients, i.e., the makeup of the calories one consumes is more important. In that light, what is the breakdown of calorie intake contributed by each class of macronutrients (carbs, proteins, fats)? Why is protein not investigated? Referring to “*the regular Ugandan diet is majorly made up of starchy roots and tubers (cassava, sweet potatoes), plantains (mostly cooking bananas), and cereals (maize, millet, sorghum)*”, these staples are low in protein, and since they form the bulk of the population’s daily diet, could the population be at risk of protein deficiency? Is stunted growth a problem in Uganda?
- Iron – is iron-deficiency a public health concern in the population? What is the prevalence of iron-deficiency anaemia?
- Same for zinc and vitamin A. Please justify why just these four nutrients are selected but not the rest. (Just because we can do something doesn't mean we should)

3. Section C: Data Description

b) Referring to Table 1, the amount of energy and most micronutrients consumed from both

market and own farm sources are far higher in year 2015 than in 2018 and 2019.

Could this anomaly affect the statistical rigour and interpretation?

4. Methods, Results and Discussions

b) “Each crop species added to those farmed with in a household was associated with 5.9 and

18.8 kilocalories (0.6 and 0.9 percentage points) added to energy sourced from own farm produce consumption or markets respectively. With regards to iron, each additional crop

species was associated with 0.4 and 0.1 milligrams (4.2 and 0.7 percentage points) added to

daily iron intake sourced from own farm produce or markets consumption respectively. With

regards to zinc, each additional crop species grown on the farm was associated with 0.3 and

0.1 milligrams (6.2 and 0.9 percentage points) added to daily zinc intake sourced from own

farm produce, and markets consumption respectively. Lastly, each additional crop species

grown on farm was associated with 1.1 and 0.6 rae_micrograms (0.3 and 0.2 percentage

points) added to daily vitamin A intake sourced from own farm produce, and markets

consumption respectively.”

What is the significance of the percentages of gains quoted above in the diets of the sample

population? Are these considered meaningful impact on nutritional status? Also, will

increment in crop diversity continue to produce nutritional gains at a linear rate or will the

effect level off? At what point will the effect level off? **We added squared FPD to detect**

**linear contributions, which are of course not continuous indefinitely, we discuss this in**

**the paper.** Check for the meaningful impact of changes by % in nutrition status. Percentages

are just used to show relative proportions – however, we quote the exact thresholds for each

micronutrient as advised by WHO and FAO, the percentages discussed in the paper, just help

us to show the proportion of the effect, with regards to average values in the sample; which

was earlier compared with thresholds to define energy and micronutrient deficient households

and those that were not deficient.

Referring to my previous question in 2(a). If the *“Average consumption of energy (2,430 kilocalories) was slightly above FAO recommended thresholds per adult”,* why *does “56*

*calories (2.3 percentage points) added for each species added to FPD”* matter? This also relates to Policy Implications.
